# A comparative study on efficacy of modified endoscopic minimally invasive treatment and traditional open surgery for primary carpal tunnel syndrome

**DOI:** 10.1186/s13018-023-03927-x

**Published:** 2023-07-18

**Authors:** Daqiang Zheng, Zhiming Wu, Sichao Cheng, Lu Li, Jianjun Chang

**Affiliations:** 1 Third Hospital of Shanxi Medical University, Shanxi Bethune Hospital, Shanxi Academy of Medical Sciences, Tongji Shanxi Hospital, Taiyuan, 030032 China; 2 Department of Orthopedics, Shanxi Bethune Hospital, Shanxi Academy of Medical Sciences, Tongji Shanxi Hospital, Third Hospital of Shanxi Medical University, Taiyuan, 030032 China

**Keywords:** Carpal tunnel syndrome, Modified transforaminal endoscopy, Modified endoscopic minimally invasive incision, Carpal tunnel release, Minimally invasive surgery

## Abstract

**Background and Objective:**

Carpal tunnel syndrome (CTS) is the most common type of median nerve entrapment neuropathy. This study aims to comparatively assess the effectiveness and clinical efficacy of modified transforaminal endoscopic minimally invasive incision of transverse carpal ligament against traditional open incision of transverse carpal ligament in the treatment of CTS.

**Method:**

The clinical data of 35 patients (57 wrists) with primary CTS treated in Shanxi Bethune Hospital, China, were retrospectively analyzed. The patients were divided into observation group (21 cases, 33 wrists) and control group (14 cases, 24 wrists), respectively, who underwent modified endoscopic minimally invasive incision of transverse carpal ligament and traditional open incision of transverse carpal ligament release. The Boston Carpal Tunnel Questionnaire (BCTQ) was assessed at for points: before the operation; 2 weeks; 1 month; and 3 months after operation. The BCTQ scores of the two groups were compared on all four points. The incidence of intraoperative and postoperative complication was used as the evaluation index. The study variables were comparatively assessed before and postoperation and also between the groups.

**Results:**

The BCTQ scores at 2 weeks, 1 month and 3 months after the operation were significantly lower than preoperative BCTQ scores (*P* < 0.005) for both the groups. There was no significant difference in BCTQ scores between the two groups at the four assessment points (*P* > 0.005). The scar size and wound healing time were significantly better with modified transforaminal endoscopic minimally invasive transverse carpal ligament incision.

**Conclusion:**

The clinical effects of both modified transforaminal minimally invasive incision of transverse carpal ligament and traditional open incision of transverse carpal ligament are significant, while the treatment efficacy of modified transforaminal minimally invasive transverse carpal ligament incision is better in terms of operation time, wound size, postoperative scar size and incision healing time.

## Introduction

Carpal tunnel syndrome (CTS) is one of the most common peripheral neuropathies and the most common type of median nerve entrapment neuropathy [1, 2]. Its incidence is relatively rare but significant ranging 3–4% in the general population and up to 8% in the working population [3]. It is more common, up to threefold, in middle-aged females and in specific working populations that work in industrial settings with forceful and repetitive wrist motion duties [4, 5].

Several pathological variations contribute to etiology, including hand osteoarthritis, rheumatoid arthritis, pregnancy, hypothyroidism, obesity and diabetes; however, CTS is mainly idiopathic and the exact mechanism and pathogenesis of CTS are not clear [1, 6, 7]. Moreover, evidence shows that exposure to high pressure and strength, repetitive work and vibration tools as well as use of certain drugs could play as risk factors for this syndrome [1, 2, 8]. The typical symptoms of CTS include nocturnal pain, tingling and numbness in the median nerve distribution area of the hand. Further progression without regular treatment may lead to atrophy of thenar muscle of the hand, disability of the hand and loss of labor ability toward the extreme [9]. This progression of the disease may seriously affect the work and sleep of patients, resulting in a decline in the quality of life of patients.

Few studies have reported that on a genetic level there are at least 16 susceptible loci for CTS, and most of these genes are expressed in the aponeuroses [10]. The involved mechanism is reportedly involved the damage of tendon cells to cartilage oligomeric matrix protein secretion [11]; however, insufficient evidence is available to support the above-mentioned theory. Concurrently, the occurrence of CTS has been confirmed to be significantly associated with the incidence of heart diseases. It is known that CTS can significantly increase the risk of myocardial amyloidosis and heart failure, as well as the risk of progression of cardiovascular diseases toward the end stage [12, 13]. Studies have exhibited that long-term compression of the median nerve can cause local loss of function mediated by myelinated and unmyelinated sensory axons [14].

Carpal tunnel release (CTR) is the standard surgical treatment option for CTS and is among the most commonly used hand surgery procedures worldwide [6]. A recently published study reported that CTR is performed on 1.9% of men and 4.1% of women during their lifetimes [6]. It also reported that hand osteoarthritis and obesity are among the common risk factors of CTR. To operate the traditional open technique, surgeons perform a traditional or mini-incision. In both technique, a longitudinal incision extending between the proximal end of the palmar crest and the distal end of the transverse carpal ligament (TCL) is done to provide a complete exposure of the median nerve [15, 16]. The landmarks for the traditional open incision are the radial border of the hypothenar muscle, where the incision with 5 cm of length, should be started and extended proximally till the distal wrist crease. The mini-incision technique involves a longitudinal incision extending between the mid-palm and the most proximal portion of the palm or a transverse incision, 2 cm in length, on the ulnar side of the wrist stripes [16, 17]. Although traditional incision access offers a complete exposure of the median nerve, it is usually associated with the risk of scar sensitivity, scar tissue and a possible flexion contracture in the wrist. These complications impose adverse effects on the surgery outcome including impaired and delayed functional recovery [18, 19]. The mini-incision technique reduces some of these complications, but the main issues of both techniques are scar pain, increased complexity and elevated chance of incomplete transverse ligament release. To improve the surgical techniques, less invasive techniques such as the mini-palmar incision surgery or endoscopic-assisted release have been proposed [9, 20, 21].

In developing and adopting a novel and less invasive CTR procedure, the ultimate objective is shortening surgical time, reducing complications such as the length of the incision and postoperative scar, faster functional recovery and faster return to work and activities in daily living. Despite these premises, all the techniques mentioned above could determine several complications, such as vascular, nerve and tendon damages, or the incomplete release of the transverse ligament, leading to recurrence of the CTS.

Different therapeutic methods have been proposed and developed for CTS; the two main groups of them are general conservative treatments and surgical treatments [2, 7, 22, 23]. Generally, conservative treatments include acupuncture, electrotherapy and pharmaceutical interventions. However, there is no literature suggesting a definitive treatment or a combination of treatment measures which leads to a good prognosis [22]. Conservative treatment can only delay the progression of the disease and relieve the symptoms temporarily, but cannot stop the disease progression. Previous studies have shown that the release of carpal tunnel and the re-innervation of the skin associated with carpal tunnel are significantly related to the improvement of symptoms and functions [24]. The evidence shows that surgical technique offers unique advantages and is still an important and effective treatment for moderate to severe CTS [25, 26]. The traditional surgical treatment involves open release of transverse carpal ligament, which is still the standard operation because of its significant therapeutic effect [27]. However, with the development of new equipment, the traditional open incision treatment is gradually being replaced by minimally invasive techniques which have shorter operation time, less trauma, smaller postoperative scar and shorter wound healing time [28]. This study aims to investigate the efficacy and highlight the characteristics and advantages of modified transforaminal minimally invasive incision of transverse carpal ligament and compare it those of the traditional open transverse carpal ligament release, by providing statistical comparison between patients undergoing these procedures separately.

## Methods

### Study design and setting

This retrospective clinical observational study was conducted on the clinical data of 35 patients (57 wrists) with primary CTS treated in Shanxi Bethune Hospital, China, between June 2021 and March 2022. This study aimed to comparatively assess the efficacy of modified transforaminal endoscopic minimally invasive of transverse carpal ligament incision against the traditional open incision of transverse carpal ligament release. Convenience sampling strategy was used to choose the clinical cases. During the study, the Boston Carpal Tunnel Questionnaire (BCTQ) was recorded at four points: before operation, 2 weeks after operation, 1 month and 3 months after operation. The BCTQ scores were then compared pre- and postoperation in each group and also between the two groups. The improvement of postoperative symptoms, functional recovery and the occurrence of complications during and after operation were taken as evaluation indexes. All the patients were treated and followed up conservatively for more than 3 months, but their symptoms were not apparently relieved. After admission, the patients’ medical histories were collected in detail and a thorough physical examination was conducted. While collecting the medical history, special attention was given to check whether the patient has a history of gout, diabetes, hypothyroidism, acromegaly, tumor, pregnancy, etc. During physical examination, it was also assessed that whether there is any trauma, edema, subcutaneous tumor, etc., around the wrist joint. Before operation, MRI of the affected wrist joint was performed uniformly, and diabetes, gout and hypothyroidism were excluded [29, 30].

### Data collection

The inclusion criteria of the study were patients who had obvious symptoms of median nerve compression, symptoms consistent with signs and poor curative effect after standardized conservative treatment for more than 3 months.

The exclusion criteria of the study were as follows: presence of secondary CTS (secondary carpal tunnel syndrome caused by diabetes, gout, hypothyroidism, acromegaly, tumor, pregnancy, edema, trauma and other wrist occupying lesions) or other diseases (cubital tunnel syndrome, thoracic outlet syndrome, cervical spondylosis) in patients, which affect the curative effect evaluation and is not conducive to intra-group and inter-group comparison, presence of mental disorders, as they are not compliant with preparing to evaluate the severity of illness and postoperative curative effect, and presence of serious co-morbidities which may interfere with the outcome of the procedure and a reliable evaluation of the efficacy of the operation cannot be deduced.

### Surgical procedures

#### Modified endoscopic minimally invasive incision

**Fig. 1:**
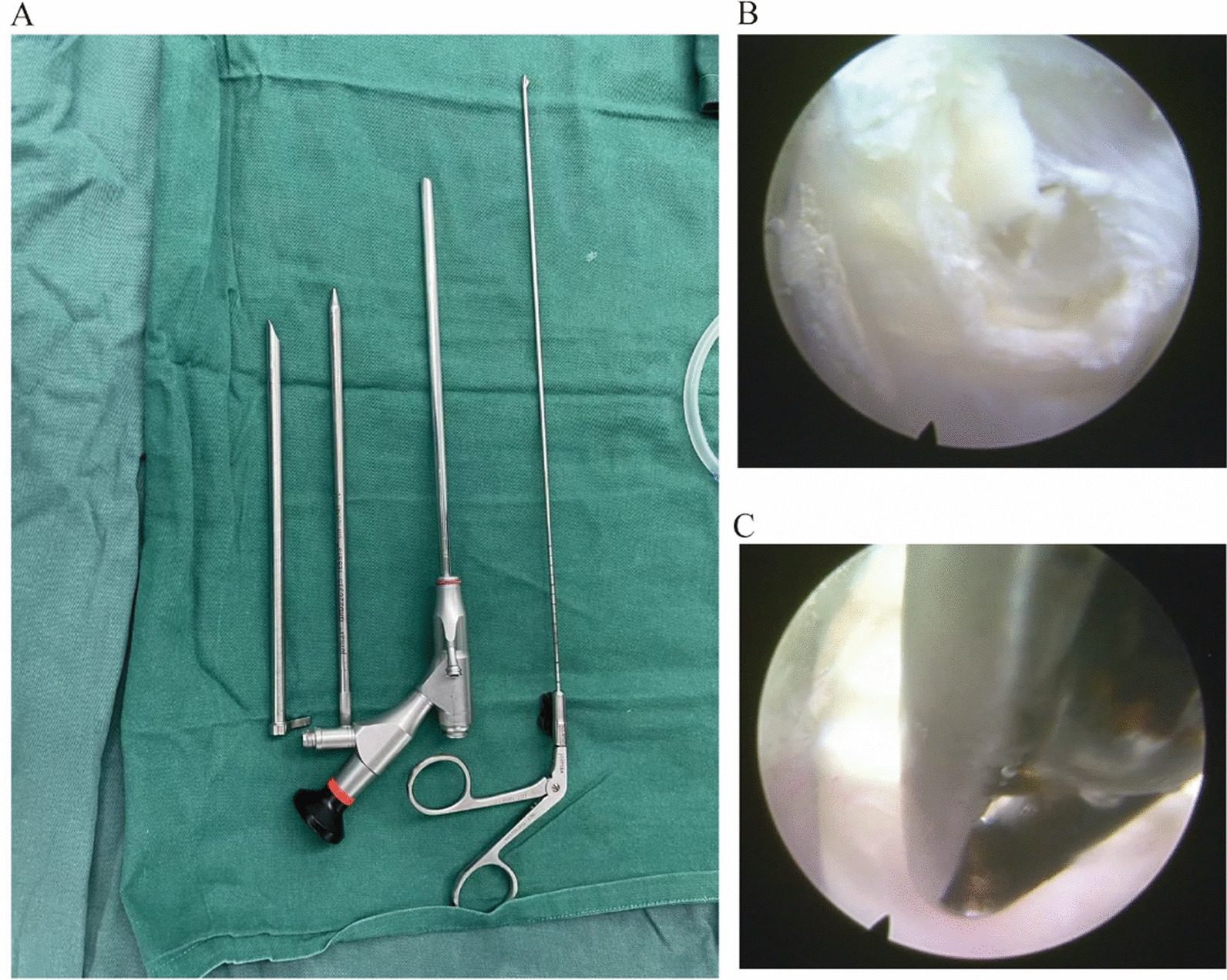
The surgical instruments required for the modified minimally invasive endoscopic procedure and the steps to be taken during the procedure. **A** The surgical instruments required for a modified minimally invasive endoscopic procedure. **B**, **C** Images of the steps to be taken in a modified minimally invasive endoscopic procedure

**Fig. 2:**
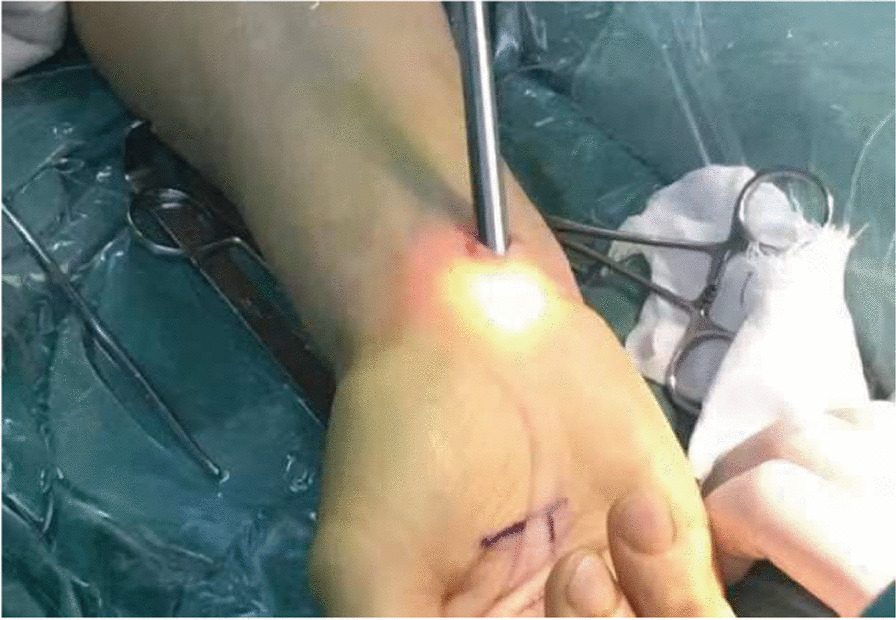
Images of an endoscopic minimally invasive incision endoscopic procedure in progress

**Fig. 3:**
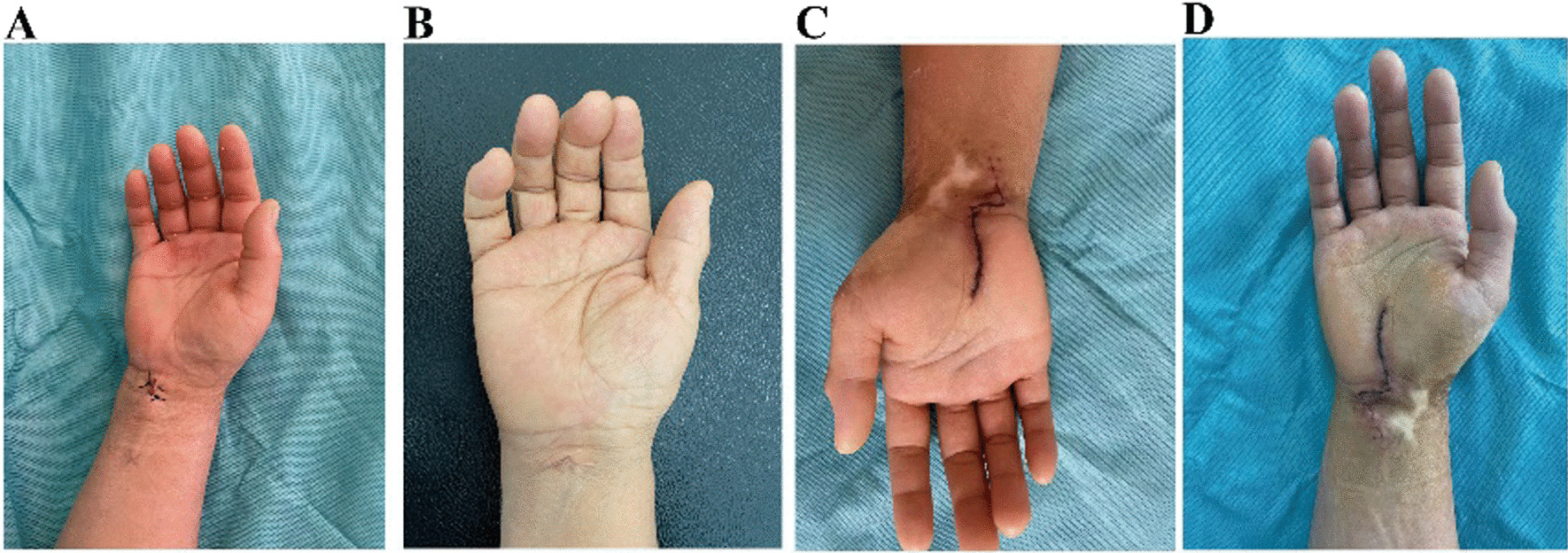
Comparison of incision conditions between the observation group and the control group 1 week after surgery. **A** Incision in the observation group 1 day after surgery. **B** Incision in the control group 1 day after surgery. **C** Incision in the observation group 1 week after surgery. **D** Incision in the control group 1 week after surgery

To perform appropriate surgical procedures in both techniques, the surgical instruments must be appropriate for a short incision (Fig. 1A). The ulnar longitudinal incision of about 1 cm was made of the proximal palmaris longus tendon of the affected wrist. The skin, subcutaneous tissue and deep fascia were cut layer by layer, and the gap between the palmaris longus tendon and flexor tendon was blunted. A soft tissue expander was placed between the far and near wrist striations, gently rotated into the working channel, and placed into the working channel (Fig. 2). The endoscopic imaging system was debugged under the stage until the image appeared clear and was washed with saline continuously. Under the endoscope, the proximal end of the transverse carpal ligament was exposed, and the working sleeve was sneaked to the distal end under the microscope. Following the complete exposure of the transverse carpal ligament, the transverse carpal ligament was cut with basket forceps (Fig. 1B, C), the working channel was gently rotated to move forward, and the transverse carpal ligament was slowly cut to the distal end under the microscope until the transverse carpal ligament incision. Simultaneously, the degenerative fatty tissue was cleaned to explore the median nerve under the microscope. Finally, the patient's incision was recorded 1 day after surgery and 1 week afterward (Fig. 3A, B).

#### Open release of transverse carpal ligament

Open release of transverse carpal ligament was performed after brachial plexus anesthesia or general anesthesia. The transverse carpal ligament was selected as the center for incision, and an S-shaped incision was made from the distal end to the palm to the proximal end to the distal forearm, to fully expose the transverse carpal ligament. A longitudinal incision was made to explore and release the epicranium of median nerve. Finally, the incision was stitched layer by layer to achieve surgical closure. Finally, the patient's incision was recorded 1 day after surgery and 1 week afterward (Fig. 3C, D).

### Postoperative treatment

After the operation, swelling and nutritional nerve treatment were given and the patients were instructed to perform functional exercises to avoid adhesion in the operation area which may affect the curative area [31].

### Assessment variables

We used the Boston Carpal Tunnel Questionnaire (BCTQ) to score the symptom dimension and functional dimension [23]. The BCTQ was recorded at four points: before operation; the 2nd week after operation; the 1st month after operation; and the 3rd month after operation. The BCTQ of these four instances was then compared between the two groups. At the same time, the scar size and the occurrence of scar pain were observed at the 2nd week, 1st month and 3rd month after operation.

### Statistical analysis

Statistical Package for Social Sciences (SPSS) (IBM SPSS Statistics for Windows, version 22.0) was used for all statistical analyses in this study. The assessment data were tested for normality by the Shapiro–Wilk test. The variables with normal distribution were expressed as mean ± standard deviation (*x* ± *s*), and independent samples *t* test was employed for inter-group comparisons. The enumeration data, such as general information about patients, were expressed as percentages or proportions, and the comparison was analyzed by the Chi-square test or Fisher's exact probability method. All statistical tests were two-sided, and *P* < 0.05 was considered statistically significant.

## Results

This study gathered the clinical data of 35 patients (57 wrists) with primary CTS who were treated in Bethune Hospital of Shanxi Province, from June 2021 to March 2022. The patients were divided into observation group, 21 cases (33 wrists) treated with minimally invasive surgery under modified intervertebral foramen, and control group, 14 cases (24 wrists) treated with open transverse carpal ligament release. Participants in the observation group comprised of 8 males and 13 females, with an average age of 49.76 ± 6.76 (SD) years. There were 9 patients with unilateral primary CTS, and 12 patients with bilateral primary CTS. In the control group (14 cases with 24 wrists), there were 5 males and 9 females, ranging from ages 34 to 60 years, with an average age of 48.50 ± 8.28 years. There were 4 patients with unilateral primary CTS and 10 patients with bilateral primary CTS in the control group.

### Demographics and general clinical parameters

There was no significant difference in demographic data between the observation group and the control group, including age, sex and lesion range (*P* value > 0.05) (Table 1).

### BCTQ scores in the observational group at different intervals

Intra-group comparison of observation group showed that BCTQ scores of observation group at the 2nd week, 1st month and 3rd month after operation were significantly lower than the preoperative BCTQ scores (*P* < 0.05) (Table 2).

**Table 1:** Comparison of general data between observation group and control group (age, sex and lesion)

	Age (year)	Gender (male/female, example)	Unilateral and bilateral (single/double, example)
Observation group	49.76 ± 6.76	7/14	9/12
control group	48.50 ± 8.28	5/9	4/10
*T* value	0.494	~	~
*P* value	0.624	1.000	0.488

**Table 2:** Comparison of BCTQ scores between the observation group at 2nd week, 1st month and 3rd month after operation and before operation (*x* ± *s*) (*n* = 21)

Time	Preoperative		2 weeks after operation		1 month after operation		3 months after operation	
	Symptom severity scale	Functional status scale	Symptom severity scale	Functional status scale	Symptom severity scale	Functional status scale	Symptom severity scale	Functional status scale
Value	35.71 ± 4.59	19.28 ± 2.98	18.38 ± 2.67	16.09 ± 1.76	19.24 ± 1.70	14.14 ± 1.39	19.48 ± 3.11	10.86 ± 1.28
*T* value			14.94	4.22	15.41	7.16	13.41	11.90
*P* value			0.00	0.00	0.00	0.00	0.00	0.00

### BCTQ scores in the control group at different intervals

In the control group, the BCTQ scores at the second week, one month and three months after operation were significantly lower than those before operation (*P* < 0.05) (Table 3).

**Table 3:** Comparison of BCTQ scores at 2 weeks, 1 month and 3 months after operation in the control group and those before operation (*x* ± *s*) (*n* = 14)

Time	Preoperative		2 weeks after operation		1 month after operation		3 months after operation	
	Symptom severity scale	Functional status scale	Symptom severity scale	Functional status scale	Symptom severity scale	Functional status scale	Symptom severity scale	Functional status scale
Value	35.28 ± 4.94	20.21 ± 3.68	18.78 ± 2.64	16.57 ± 1.83	19.14 ± 2.07	14.43 ± 1.70	18.86 ± 3.03	11.29 ± 1.59
*T* value			11.03	3.31	11.28	5.34	10.61	8.33
*P* value			0.00	0.00	0.00	0.00	0.00	0.00

### Comparison of BCTQ scores between observational and control at different intervals

There was no significant statistical difference (*P* > 0.05), as shown in Table 4, between the observational and control groups in the BCTQ scores at different intervals: before operation; 2 weeks after operation, 1 month after operation; and 3 months after operation.

**Table 4:** Comparison of BCTQ scores of observation group before operation, 2 weeks after operation, 1 month after operation and 3 months after operation compared with those of control group before operation, 2 weeks after operation, 1 month after operation and 3 months after operation (*x* ± *s*)

Group	Number of cases	Preoperative		2 weeks after operation		1 month after operation		3 months after operation	
		Symptom severity scale	Functional status scale	Symptom severity scale	Functional status scale	Symptom severity scale	Functional status scale	Symptom severity scale	Functional status scale
Observation group	21	35.71 ± 4.59	19.28 ± 2.98	18.38 ± 2.67	16.09 ± 1.76	19.23 ± 1.70	14.14 ± 1.39	19.48 ± 3.11	10.86 ± 1.28
Control group	14	35.28 ± 4.94	20.21 ± 3.68	18.79 ± 2.64	16.57 ± 1.83	19.14 ± 2.07	14.43 ± 1.70	18.857 ± 3.03	11.286 ± 1.59
*T* value		0.26	− 0.82	− 0.44	0.77	0.15	− 0.55	0.58	− 0.88
*P* value		0.79	0.42	0.66	0.44	0.88	0.60	0.56	0.38

### Operation time and scar pain

The operation time of the observational group was significantly shorter, *P* < 0.05, than that of the control group. However, there was no significant difference between the two groups in the incidence of scar pain during 3 months after the operation (Table 5).

**Table 5:** Comparison of operation time (unilateral) and scar pain incidence within 3 months after operation between the observation group and the control group

	Operation time	Incidence of scar pain
	(min)	(cases of scar pain/total cases in the group)
Observation group	33.57 ± 4.21	1/21
Control group	56.78 ± 7.93	3/14
*T* value	− 11.28	~
*P* value	0.000	0.300

## Discussion

CTS is one of the most common peripheral neuropathies, characterized by a series of symptoms and pathophysiological changes that gradually deteriorate the quality of life of patients. Currently, open carpal tunnel release is still considered the gold standard procedure for the treatment of CTS, which is, however, ineffective in conservative treatment. Due to high risk of surgery this procedure has been criticized by doctors as well as the patients. During the current times, innovation of new surgical equipment and applications are changing the face of all kinds of surgical operations toward more accurate and minimally invasive techniques [27]. This comes with the advantages of early recovery of hand function and the lower recurrence rate.

The basic principle of the operation for CTS is to increase the volume of the carpal tunnel and reduce the pressure on the median nerve by breaking the transverse carpal ligament [1, 30, 32, 33]. Shin et al. have shown that endoscopic single transection of the transverse carpal ligament is effective in the treatment of CTS, which is comparable to open carpal tunnel release in terms of postoperative symptom improvement, pain score and functional recovery [34]. Compared to endoscopic surgery, modified transforaminal endoscopic surgery has the advantages of clearer operative field, simpler operation, shorter learning time, and less possibility of damaging the surrounding soft tissue during operation. Yu et al. [35] reported that in spinal surgery, transforaminal endoscopic surgery is superior to endoscopic spinal surgery in terms of average length of stay and Oswestry disability index (ODI) score.

Open carpal tunnel release is one of the most common and successful operations for the treatment of primary CTS. The common complications are postoperative infection, scar pain and soft tissue adhesion in the operation area. Our study had 1 patient in the observation group with postoperative scar pain, 3 patients in the control group with scar pain, and 35 patients had no postoperative infection and soft tissue adhesion. When comparing the two procedures, both seem to have the same advantages and similar characteristics, such as incision size and operation time, etc.; however, arthroscopic transverse carpal ligament disruption has a better control over the complications when compared to open carpal tunnel release.

A comparison of the study variables before and after surgery in each group, as well as a comparison of the observation and control groups, revealed that the modified minimally invasive transverse carpal ligament dissection for median nerve release was more effective and more efficient than the traditional open transverse carpal ligament release in terms of operative time, wound size, postoperative scar size and incision healing time. Translated with www.DeepL.com/Translator (free version). The median nerve release was superior. A study by Larsen et al. [36] showed that the time required for patients to return to work and to normal daily life was significantly shorter in those who were operated with minimally invasive endoscopic transverse carpal ligament incision and median nerve release, compared to those who were operated with traditional open transverse carpal ligament release. At the same time, in a satisfaction survey report, Kang et al. [37] showed that most patients preferred endoscopic technology, mainly because they were worried about scar retention and/or pain from the incision.

Transforaminal endoscopy is a recognized minimally invasive technology, which integrates a light source lighting system, image acquisition, amplification system, and continuous water irrigation system. The existence of light source lighting system makes the surgical field of vision bright, while the image acquisition and amplification system plus continuous water irrigation system makes the anatomical level of the surgical field clearer, which is conducive to the operator to carry out more precise operation. Moreover, it is beneficial in reducing the probability of injury of surrounding tissues during operation and also reduces the operation risk of patients [38, 39].

The limitation of modified transforaminal endoscopic minimally invasive transverse carpal ligament incision is that the operator must master the anatomical relationship of wrist, and the clarity and accuracy of the exposure of endoscopic surgery field. Under endoscopy, the degenerative adipose tissue accumulated around the transverse carpal ligament and median nerve should be cut gradually with blue forceps, and the flocculent soft tissue floating in the operation field should be cleaned at the same time. When bleeding is seen, radio-frequency hemostasis should be applied. While operating, it is necessary to confirm that the transverse carpal ligament has been cut and that there is no accumulation of degenerative adipose tissue above the median nerve. Meanwhile, it is also necessary to avoid damaging the surrounding vascular and neural structures.

In the modified transforaminal endoscopic carpal ligament transection, blue forceps are used to separate the transverse carpal ligament. In this process, about 0.2 cm of transverse carpal ligament is removed by vertical carpal tunnel clamp, which is also one of the differentiating points between the 2 operations. The significance of this operation has not been well reported; however, we found out that this operation can further increase the volume of the carpal canal and the median nerve is also released to a greater extent.

Transforaminal endoscopy is not a new technique and spinal surgeons have always been familiar with this concept, thus, this equipment is easily available in most hospitals. Moreover, transforaminal endoscopy has gained more success over the past few years which has motivated surgeons to gain experience, increasing the application of technology of intervertebral foramen mirror.

Coupled with the experience of traditional open transverse carpal ligament release, it is easier learning the modified minimally invasive surgery of transverse carpal ligament [17, 40]. To practice this technique as a beginner, the surgeon should be familiar with the local anatomy of the wrist, and must have good three-dimensional spatial imagination, along with having the experience of traditional open release of transverse carpal ligament. The operation of suspension of both hands and clearly exposing the surgical field can ensure complete hemostasis and pre-hemostasis, which requires experienced guidance and teaching.

The minimally invasive surgery of transverse carpal ligament under intervertebral foraminal endoscope, discussed in this study, not only eliminates the degree and scope of postoperative scar, but also significantly reduces the probability of postoperative scar pain. However, data confirm that compared with traditional incision, both techniques have achieved the disconnection of transverse carpal ligament and the release of median nerve to a similar extent and there is no significant difference in the treatment effect.

Our findings showed that modified transforaminal endoscopic minimally invasive incision of carpal transverse ligament is a unique CTS treatment that should be considered as a better treatment option, which requires more research in the field. In order to ensure early recovery of postoperative function, we generally encourage patients to initiate functional exercise of wrist joint on the second day after operation, so as to achieve a better therapeutic effect.

## Conclusion

To sum up, the effect of modified transforaminal endoscopic minimally invasive carpal transverse ligament disruption in the treatment of primary CTS is even though similar to that of the traditional incision treatment, the treatment effect in terms of operation time, wound size, postoperative scar size, incision healing time and other aspects are better with the former. It is imperative to promote the use of this technique as this will provide CTS patients with an option of minimally invasive and more accurate procedure with lesser surgical risk and complications.

## Data Availability

The data that support the findings of this study are available on request from the corresponding author.
